# Delays in presentation of intussusception and development of gangrene in Zimbabwe

**DOI:** 10.11604/pamj.supp.2021.39.1.21301

**Published:** 2021-07-28

**Authors:** Dennis Mazingi, Eleanor Burnett, Hilda Angela Mujuru, Kusum Nathoo, Jacqueline Tate, Jason Mathiu Mwenda, Goitom Weldegebriel, Portia Manangazira, Arnold Mukaratirwa, Umesh Parashar, Taurai Zimunhu, Bothwell Anesu Mbuwayesango

**Affiliations:** 1 Department of Surgery, University of Zimbabwe, Zimbabwe,; 2 Centers for Disease Control and Prevention, Atlanta, USA,; 3 Department of Pediatrics and Child Health, University of Zimbabwe, Zimbabwe,; 4 World Health Organization (WHO) Regional Office for Africa, Brazzaville, Republic of Congo,; 5 Epidemiology and Disease Control, Ministry of Health and Child Care, Harare, Zimbabwe

**Keywords:** Intussusception, gangrene, intestinal obstruction, delay, developing countries, global paediatric surgery

## Abstract

**Introduction:**

prompt diagnosis and treatment are considered key to successful management of intussusception. We examined pre-treatment delay among intussusception cases in Zimbabwe and conducted an exploratory analysis of factors associated with intraoperative finding of gangrene.

**Methods:**

data were prospectively collected as part of the African Intussusception Network using a questionnaire administered on consecutive patients with intussusception managed at Harare Children´s Hospital. Delays were classified using the Three-Delays-Model: care-seeking delay (time from onset of symptoms to first presentation for health care), health-system delay (referral time from presentation to first facility to treatment facility) and treatment delay (time from presentation at treatment facility to treatment).

**Results:**

ninety-two patients were enrolled from August 2014 to December 2016. The mean care-seeking interval was 1.9 days, the mean health-system interval was 1.5 days, and the mean treatment interval was 1.1 days. Mean total time from symptom onset to treatment was 4.4 days. Being transferred from another institution added 1.4 days to the patient journey. Gangrene was found in 2 (25%) of children who received treatment within 1 day, 13 (41%) of children who received treatment 2-3 days, and 26 (50%) of children who received treatment more than 3 days after symptom onset (p = 0.34).

**Conclusion:**

significant care-seeking and health-system delays are encountered by intussusception patients in Zimbabwe. Our findings highlight the need to explore approaches to improve the early diagnosis of intussusception and prompt referral of patients for treatment.

## Introduction

Intussusception is an enteric invagination into an adjacent segment of bowel. Some intussusception cases have been associated with infection with various enteric viruses causing Peyer´s patch hypertrophy [1, 2]. This assertion was bolstered by some studies finding a seasonal pattern of intussusception cases [3, 4]. A slightly increased risk of intussusception of 1 to 6 excess cases per 100,000 vaccinated infants has been observed following rotavirus vaccination in clinical trials in high- and middle-income countries [5]; however, no association was found between rotavirus vaccine and intussusception in a multi-country analysis in sub-Saharan Africa [6]. Intussusception is the most common cause of childhood intestinal obstruction in Zimbabwe [3], and is also the most frequently encountered paediatric surgical emergency [3]. This is similar to the experience in other African countries [7]. It was found to be the most common cause of childhood intestinal obstruction in Nigeria and of acute mechanical obstruction in children in Niger [8, 9].

Intussusception is managed surgically, with manual reduction or resection, or nonoperatively by air, hydrostatic or contrast enema. In Africa rates of surgical intervention are higher than for non-operative reduction [6, 7, 10]. Ekenze *et al*. reported that in south eastern Nigeria surgical management was performed routinely in cases of intussusception [11]. In contrast, 81% of intussusception patients in a study in Europe had non-operative reduction [12].

Delays in presentation and treatment of serious surgical diseases, including intussusception, are common in low-resource countries due to limited access to care [7, 10, 13, 14]. In a study from Nigeria only 7.7% of patients presented within 24 hours of onset of intussusception symptoms [15]. Late presentation of intussusception cases is considered a risk factor for gangrene and death, increasing the need for surgery [16-18] and predicting the failure of non-operative reduction [16, 19-20]. It also increases the chances of sepsis, multiple organ dysfunction and death. [21, 22] In this analysis we describe the time intervals from onset of symptoms to definitive treatment of infants with intussusception in Zimbabwe. As an exploratory analysis, we considered the relationship between delayed presentation and gangrene.

## Methods

### Patient population

**Figure 1:**
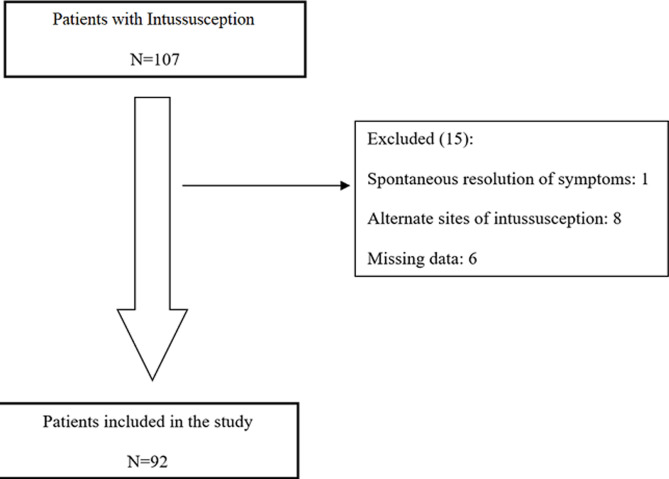
study participants flow chart

All patients < 12 months old admitted and treated for intussusception at Harare Children´s Hospital from August 2014 to December 2016 and enrolled as part of the African Intussusception Surveillance Network were included in this analysis. Patients were included if they fulfilled level 1 of the Brighton Collaboration Intussusception Working Group criteria of diagnostic certainty [23]. For this analysis, patients were excluded if they did not have an ileocolic intussusception (Figure 1). Non-ileocolic intussusception is frequently caused by a distinct lead point [24, 25], which would confound the effect of embryological mechanical factors.

### Study setting

The study was performed at Harare Children´s Hospital, a public, teaching referral hospital.

### Data collection

Data were collected using a structured questionnaire on admission and during hospital stay. Information regarding age, sex, home address, pertinent dates in the referral journey, method of definitive treatment, intraoperative findings, and procedure performed was collected. Patient codes were used to anonymize the data. Patients with missing time interval and intraoperative data were excluded from statistical analysis (Figure 1).

### Description of surgical procedure

Patients were operated by the paediatric surgical team of 10 experienced surgeons and surgical trainees at Harare Children´s Hospital paediatric theatre. The surgical procedure was performed as per institutional standard and involved initial exploratory laparotomy with an attempt at reduction made if bowel was assessed to be viable. Bowel was considered to be viable if bowel had good colour, contractility and consistency as well as strong mesenteric pulsations. Bowel was resected with primary anastomosis if it was judged to be gangrenous, based on these four parameters. The viability of the unresected intestines was confirmed by post-operative follow-up. Gangrene of resected intestines was corroborated on histological examination of resected specimens which is performed routinely for all resections.

### Definitions of time intervals

The time from symptom onset to definitive management was split into three time intervals using a modification of Three Delays Model [26]. This includes: care-seeking interval, health-system interval and treatment interval. Composite intervals were added to this model as described below. The care-seeking interval was calculated as the time in days from the date of first symptoms to the date of first contact with the health system at a conventional medical institution. The health-system interval was calculated from the date of first contact with the health system until the date of admission to Harare Children´s Hospital. Treatment interval was calculated as the time in days from the date of admission at Harare Children´s Hospital to the date of definitive management. Total time to hospital (TTH) was calculated as the time in days from the date of symptom onset to the date of admission at Harare Children´s Hospital, in cases where the child was not transferred from another facility and the first contact with the healthcare system was Harare Children´s Hospital, the care-seeking interval and time to hospital were equal. Total time to treatment (TTT) was calculated from the date of symptom onset to date of definitive treatment.

### Statistical analysis

We used descriptive statistics to describe the demographic characteristics and the patient journey time intervals. Sample means, and standard deviations were calculated for each interval. A dependent t-test was used to determine whether the care-seeking interval and health-system intervals were significantly different from one another. We used chi-square or Fisher´s exact tests to investigate whether a relationship existed between time to hospital; time to treatment; referral status and the intraoperative finding of gangrene. P-values of < 0.05 were considered significant.

**Ethical approval:** ethical approval for this publication has been waived by the Medical Research Council of Zimbabwe.

**Disclaimer:** the findings and conclusions of this report are those of the authors and do not necessarily represent the official position of the US Centers for Disease Control and Prevention.

## Results

### Demographics

Ninety two (92) patients with intussusception were included in this analysis. 59 (64%) were male with a male to female ratio of 1.8:1. The median age was 6 months and interquartile range was 5-9 months. All patients were treated with surgery and 41 (45%) developed gangrene.

### Geographic factors

**Figure 2:**
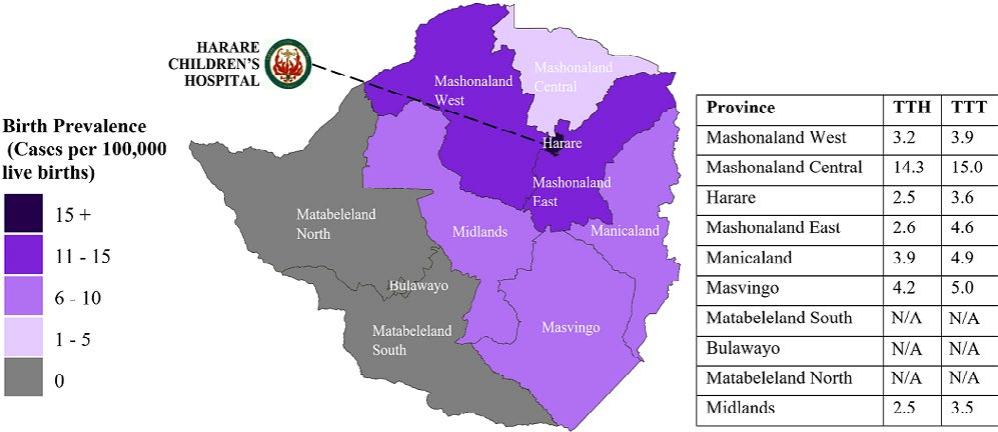
home addresses of patients with intussusception: a map of Zimbabwe with level one administrative boundaries (provinces) showing the distribution of intussusception cases as cases per 100,000 live births; the location of Harare Children´s Hospital is shown; mean time to hospital (TTH) and time to treatment (TTT) in days for each province are also shown

Home addresses were used to determine where patients lived at the time of illness onset. Figure 2 shows the distribution of patients according to home address in Zimbabwe and the mean delay for each province. The prevalence ranged from 25.8 per 100,000 live births in Harare to 3.3 per 100,000 live births in Mashonaland Central. There were no cases admitted to Harare Children´s Hospital from the provinces of Matabeleland North, Matabeleland South, or Bulawayo for intussusception during the surveillance period. The shortest mean time to hospital was 2.5 days among children from Harare and Midlands provinces. The longest mean time to hospital was 14.3 days among children from Mashonaland Central.

### Time intervals in the patient journey

**Figure 3:**
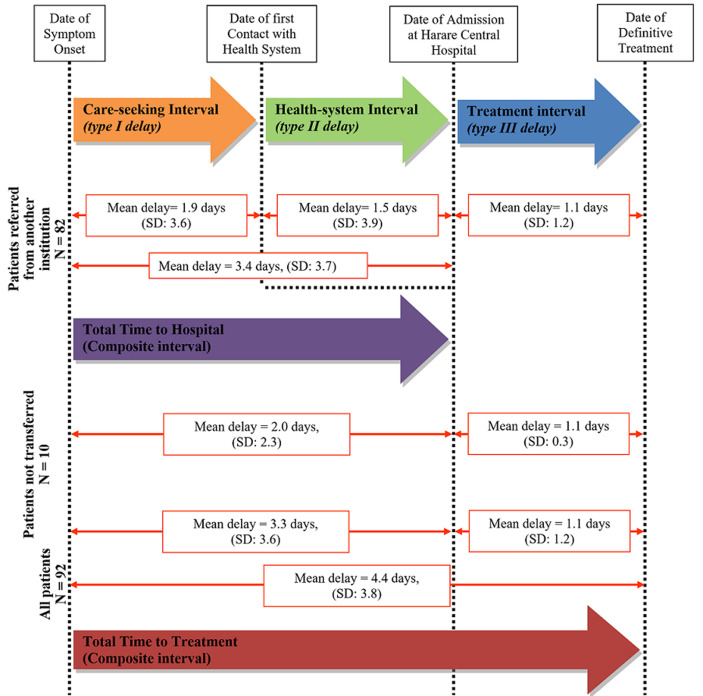
time intervals involved in the patient journey to treatment: table shows how time intervals were measured and the calculation of composite time intervals; mean time (in days) for each component of the patient journey as well as composite time intervals are shown from the results

eighty two (82) patients (89%) were transferred to Harare Children´s Hospital from another health institution and 10 patients (11%) came directly from home. Of those who were transferred from another hospital, mean care-seeking interval, health-system interval and treatment interval were 1.9 days (SD: 3.6), 1.5 days (SD: 1.9) and 1.1 days (SD: 1.2) respectively. No significant difference was observed between the care-seeking interval and health-system interval (p = 0.501). For patients admitted from home, mean care-seeking interval was 2.0 days (SD 2.3) and treatment interval was 1.1 days (SD: 0.3) (Figure 3). For all patients, the mean treatment interval was 1.1 days (SD: 1.2). Mean time to hospital was 3.3 days (SD: 3.6) and mean time to treatment was 4.4 days (SD: 3.8). Children who were transferred from another facility to Harare Children´s Hospital had an average of 1.4 days longer time to hospital compared to children who were not transferred.

### Relationship with development of gangrene

Of the patients that were transferred from another facility, 44% (n = 36) developed gangrene and 56% (n = 46) did not (p = 0.75) (Table 1). Gangrene was found intraoperatively in 38% (n = 9) of children who arrived to hospital within 1 day, 42% (n = 16) of those who arrived to hospital 2-3 days, and 53% of those who arrived at hospital more than 3 days of symptom onset (p = 0.47). Similarly, gangrene was found intraoperatively in 25% (n = 2) of children who received treatment within 1 day, 41% (n = 13) of children who received treatment 2-3 days, and 50% (n = 26) of children who received treatment more than 3 days after symptom onset (p = 0.34).

**Table 1:** relationship between transfer status, time to hospital, time to treatment with intraoperative finding of gangrene

		Gangrene (n=41)		No gangrene (n=51)		
		n	%	n	%	p-value
**Gender1**	Male	27	46	32	54	0.76
	Female	14	42	19	58	
**Age (Months)**	Median, (IQR)	7, (4-10)		6, (5-8)		
**Transferred2**	Yes	36	44	46	56	0.75
	No	5	50	5	50	
**Time to Hospital1**	≤1 day	9	38	15	63	0.47
	2 - 3 days	16	42	22	58	
	> 3 days	16	53	14	47	
**Time to treatment1**	≤ 1 day	2	25	6	75	0.34
	2 - 3 days	13	41	19	59	
	>3 days	26	50	26	50	

1Chi-square statistic; 2Fisher´s exact test

### Complications

Five patients died postoperatively due to multi-organ dysfunction. Three patients died after hospital discharge from unrelated causes. One patient required another laparotomy 1 month postoperatively for adhesive small bowel obstruction.

## Discussion

We found significant delays between the onset of intussusception symptoms and reduction among children < 12 months old in Zimbabwe. The mean care-seeking interval was slightly higher than the mean health-system interval but this difference was not statistically significant. Therefore, both intervals likely contributed equally to delays in reaching definitive treatment. The evidence to date would suggest that diagnostic delay plays a large part in late presentation rather than socioeconomic factors, which has been reported by other evaluations [27-29]. Barriers to timely care in paediatric surgery were explored by Pilkington *et al* and include transport and cost on the part of the patient as well as shortcomings in hospital infrastructure and resources [30]. The mean treatment interval was 1.1 days in our study and was comparable to guidelines for wait times in paediatric surgical patients formulated by the Canadian Paediatric Surgical Wait Times Taskforce [31]. It was also much shorter than average treatment interval in Uganda [30]. This is a surrogate quality measure and shows that, definitive management is instituted quickly once the decision has been made.

Surgery was used to manage intussusception for 100% of this study population because of lack of facilities required for enema reduction during the study period. Additionally, when duration of symptoms is more than 24 hours, surgeons may be tempted to forgo non-operative reduction because of a presumed high rate of failure in these patients. The percentage of patients who received surgery is very high when compared to the much lower rates observed in Europe (19%) [12] and Vietnam (8%) [2]. The provision of facilities for non-operative reduction should be prioritised since a sizeable percentage of patients may be amenable to this method of treatment even when they present late.

While we observed a trend toward increasing rates of gangrene with increasing intervals from intussusception onset to treatment, the results were not statistically significant likely because of our small sample size. Although some previous studies have found such a relationship [18-20], other studies have not found a relationship between duration of symptoms and success of non-operative reduction or need for surgery [17, 32-38]. Gangrene is the major reason for failure of nonoperative reduction and failure of reduction may be considered a proxy for gangrene. This suggests there may be additional factors that influence the development of gangrene. Mechanical factors have been suggested that influence the tension or pressure on mesenteric blood vessels including abnormalities of intestinal fixation [39-41]. The assertion by Brereton [42], Gil-Vargas [40] and others [43] that an excessively long, loose mesentery may be an etiological factor for intussusception is plausible. It may also protect the bowel from the development of gangrene. Furthermore, rectal protrusion of intussusception has been thought to represent an excessive delay in presentation [44, 45], but equally could reflect excessive laxity of the mesentery of normally fixed retroperitoneal structures [46]. One patient from Nigeria with rectal protrusion reported presented after 28 days and had no gangrene or perforation [47]. Similarly, in our study one patient received definitive treatment 33 days after onset of symptoms and had viable bowel requiring only manual reduction. Further research is needed in this area.

### Limitations

A major limitation of this study is that intraoperative clinical judgment was used in the determination of intestinal gangrene, which may have overestimated the presence of gangrene compared to other techniques such as fluorescence or laser Doppler ultrasound [48-50]. However, there was > 95% concordance between histological assessment and clinical judgment in this population suggesting that clinical judgment was an acceptable method for intraoperative gangrene assessment for this study. The dates of intussusception symptom onset were self-reported by each child´s caregiver and were not able to be verified. As a result, there may have been bias introduced into these findings.

The data shows a trend towards higher rates of gangrene when the pre-hospital and pre-treatment delay is longer. The inability to find a statistically significant relationship may have been related to inadequate power of the study to detect differences considering the low sample sizes in some cells. Future studies with larger sample sizes could help clarify this possibility. Because this was a single-centre study, it may not be generalizable to all of Zimbabwe. Harare Children´s Hospital is the only dedicated paediatric hospital in Zimbabwe, however a small number of patients from the south-west of the country are managed by general surgeons in the region.

## Conclusion

Time to hospital for treatment of intussusception in Zimbabwe is longer than commonly accepted benchmarks. Low sample size in this study may not have provided enough statistical power to show significant associations between gangrene and pre-hospital and pre-treatment duration although these may have existed. Advocacy and training among primary care providers to improve timeliness and accuracy of diagnosis and capacitating small peripheral health institutions as well as health education in parents to improve healthcare-seeking behaviour are potential targets for reducing delays in the pre-treatment interval. Future research should investigate mechanical factors and the morphology of the bowel in intussusception.

### What is known about this topic

- Rotavirus vaccines have been associated with an increased risk of intussusception in some high and middle income countries but not in countries in sub-Saharan Africa;
- Data on the epidemiology of intussusception in sub-Saharan African are sparse.

### What this study adds

- Intussusception rarely occurs in the first three months of life in Ethiopia when rotavirus vaccine doses are given;
- Children with intussusception who die are more likely to present later for treatment than children who survive.

## References

[1] Okimoto S, Hyodo S, Yamamoto M, Nakamura K, Kobayashi M (2011). Association of viral isolates from stool samples with intussusception in children. Int J Infect Dis.

[2] Bines JE, Liem NT, Justice FA, Son TN, Kirkwood CD, Campo M de (2006). Risk factors for intussusception in infants in Vietnam and Australia: Adenovirus implicated, but not rotavirus. J Pediatr.

[3] Mazingi DS, Mbuwayesango BA, Zimunhu T, Kumirayi L, Mahomva F, Mushonga M (2015). Seasonality and surgical management of intussusception over 10 years at Harare Children´s Hospital. Cent Afr J Med.

[4] Samad L, Cortina-Borja M, Bashir HE, Sutcliffe AG, Marven S, Cameron JC (2013). Intussusception incidence among infants in the UK and Republic of Ireland: A pre-rotavirus vaccine prospective surveillance study. Vaccine.

[5] Patel MM, López-Collada VR, Bulhões MM, De Oliveira LH, Márquez AB, Flannery B (2011). Intussusception Risk and Health Benefits of Rotavirus Vaccination in Mexico and Brazil. N Engl J Med.

[6] Tate JE, Mwenda Jason Mathiu, Armah G, Jani B, Omore R, Ademe A (2018). Evaluation of Intussusception after Monovalent Rotavirus Vaccination in Africa. N Engl J Med.

[7] Ogundoyin OO, Olulana DI, Lawal TA (2016). Childhood intussusception: Impact of delay in presentation in a developing country. Afr J Paediatr Surg.

[8] Uba AF, Edino ST, Yakubu AA, Sheshe AA (2004). Childhood intestinal obstruction in Northwestern Nigeria. West Afr J Med.

[9] Adamou H, Magagi IA, Habou O, Adakal O, Ganiou K, Amadou M (2017). Acute mechanical intestinal obstruction in children at zinder national hospital, Niger: Aetiologies and prognosis. Afr J Paediatr Surg.

[10] Bode CO (2008). Presentation and management outcome of childhood intussusception in Lagos: A prospective study. Afr J Paediatr Surg.

[11] Ekenze SO, Mgbor SO, Okwesili OR (2010). Routine surgical intervention for childhood intussusception in a developing country. Ann Afr Med.

[12] Huppertz H-I, Soriano-Gabarro M, Grimprel E, Franco E, Mezner Z, Desselberger U (2006). Intussusception Among Young Children in Europe: Pediatr Infect Dis J.

[13] Ozgediz D, Jamison D, Cherian M, McQueen K (2008). The burden of surgical conditions and access to surgical care in low-and middle-income countries. Bull World Health Organ.

[14] Ekenze SO, Mgbor SO (2011). Childhood intussusception: The implications of delayed presentation. Afr J Paediatr Surg.

[15] Talabi AO, Sowande OA, Etonyeaku CA, Adejuyigbe O (2013). Childhood intussusception in Ile-ife: What has changed?. Afr J Paediatr Surg.

[16] Reijnen JA, Festen C, van Roosmalen RP (1990). Intussusception: factors related to treatment. Arch Dis Child.

[17] Lehnert T, Sorge I, Till H, Rolle U (2009). Intussusception in children-clinical presentation, diagnosis and management. Int J Colorectal Dis.

[18] Wong CWY, Jin S, Chen J, Tam PKH, Wong KKY (2016). Predictors for bowel resection and the presence of a pathological lead point for operated childhood intussusception: A multi-center study. J Pediatr Surg.

[19] Fike FB, Mortellaro VE, Holcomb GW, Peter SDS (2012). Predictors of failed enema reduction in childhood intussusception. J Pediatr Surg.

[20] Chung JL, Kong MS, Lin JN, Wang KL, Lou CC, Wong HF (1994). Intussusception in infants and children: risk factors leading to surgical reduction. J Formos Med Assoc Taiwan Yi Zhi.

[21] Bhatnagar BNS, Sharma CLN, Gautam A, Kakar A, Reddy DCS (2004). Gangrenous sigmoid volvulus: a clinical study of 76 patients. Int J Colorectal Dis.

[22] Fevang BT, Fevang J, Stangeland L, Søreide O, Svanes K, Viste A (2000). Complications and death after surgical treatment of small bowel obstruction. Ann Surg.

[23] Bines JE, Ivanoff B, Justice F, Mulholland K (2004). Clinical Case Definition for the Diagnosis of Acute Intussusception. J Pediatr Gastroenterol Nutr.

[24] Eklöf OA, Johanson L, Löhr G (1980). Childhood intussusception: Hydrostatic reducibility and incidence of leading points in different age groups. Pediatr Radiol.

[25] Ein SH (1976). Leading points in childhood intussusception. J Pediatr Surg.

[26] Thaddeus S, Maine D (1994). Too far to walk: Maternal mortality in context. Soc Sci Med.

[27] Justice FA, Auldist AW, Bines JE (2006). Intussusception: Trends in clinical presentation and management. J Gastroenterol Hepatol.

[28] Simon RA, Hugh TJ, Curtin AM (1994). Childhood Intussusception in a Regional Hospital. Aust N Z J Surg.

[29] Stringer MD, Pledger G, Drake DP (1992). Childhood deaths from intussusception in England and Wales, 1984-9. BMJ.

[30] Pilkington M, Situma M, Winthrop A, Poenaru D (2018). Quantifying delays and self-identified barriers to timely access to pediatric surgery at Mbarara Regional Referral Hospital, Uganda. J Pediatr Surg.

[31] Wright JG, Li K, Seguin C, Booth M, Fitzgerald P, Jones S (2011). Development of pediatric wait time access targets. Can J Surg.

[32] McDermott VG, Taylor T, Mackenzie S, Hendry GM (1994). Pneumatic reduction of intussusception: clinical experience and factors affecting outcome. Clin Radiol.

[33] Tareen F, Ryan S, Avanzini S, Pena V, Mc Laughlin D, Puri P (2011). Does the length of the history influence the outcome of pneumatic reduction of intussusception in children?. Pediatr Surg Int.

[34] Okuyama H, Nakai H, Okada A (1999). Is barium enema reduction safe and effective in patients with a long duration of intussusception?. Pediatr Surg Int.

[35] Wong CW, Chan IH, Chung PH, Lan LC, Lam WM, Department of Surgery, The University of Hong Kong, Queen Mary Hospital, Pokfulam, Hong Kong (2015). Childhood intussusception: 17-year experience at a tertiary referral centre in Hong Kong. Hong Kong Med J.

[36] Kaiser AD, Applegate KE, Ladd AP (2007). Current success in the treatment of intussusception in children. Surgery.

[37] Gorenstein A, Raucher A, Serour F, Witzling M, Katz R (1998). Intussusception in children: reduction with repeated, delayed air enema. Radiology.

[38] Shapkina AN, Shapkin VV, Nelubov IV, Pryanishena LT (2006). Intussusception in children: 11-year experience in Vladivostok. Pediatr Surg Int.

[39] Shafik AA, Shafik A, Asaad S, Wahdan M (2010). A study of an anatomic-physiological cecocolonic sphincter in humans. Clin Anat.

[40] Gil-Vargas M, Sol-Meléndez AK, Miguel-Sardaneta ML (2016). Is intestinal malrotation the cause of intussusception? Waugh´s syndrome, case report. Cir Cir.

[41] Mazingi D, Mbanje C, Muguti GI, Zimunhu T, Mbuwayesango B (2019). Volvulus of the ascending colon due to failure of zygosis: A case report and review of the literature. Int J Surg Case Rep.

[42] Brereton RJ, Taylor B, Hall CM (1986). Intussusception and intestinal malrotation in infants: Waugh´s syndrome. BJS.

[43] Inan M, Basaran UN, Ayvaz S, Pul M (2004). Waugh´s syndrome: report of two cases. J Pediatr Surg.

[44] Tianyi F-L, Kadia BM, Dimala CA, Agbor VN (2017). Delayed diagnosis of transanal prolapse of an ileo-colic intussusception in a 10-month-old infant in rural Cameroon: a case report. BMC Res Notes.

[45] Ray A, Mandal KC, Shukla RM, Roy D, Mukhopadhyay B, Bhattacharya M (2012). Neglected Intussusception Presenting as Transanal Prolapse of Small Bowel. Indian J Pediatr.

[46] Frydman J, Ben-Ishay O, Kluger Y (2013). Total ileocolic intussusception with rectal prolapse presenting in an adult: a case report and review of the literature. World J Emerg Surg.

[47] Ameh EA, Mshelbwala PM (2008). Transanal protrusion of intussusception in infants is associated with high morbidity and mortality. Ann Trop Paediatr.

[48] Liao X, She Y, Shi C, Zhang Z, Li M (1994). Comparison of methods for the determination of viability of ischemic rabbit intestine. Pediatr Surg Int.

[49] Cooperman M, Martin EW, Carey LC (1980). Evaluation of ischemic intestine by Doppler ultrasound. Am J Surg.

[50] Bulkley GB, Zuidema GD, Hamilton SR, O´Mara CS, Klacsmann PG, Horn SD (1981). Intraoperative determination of small intestinal viability following ischemic injury: a prospective, controlled trial of two adjuvant methods (Doppler and fluorescein) compared with standard clinical judgment. Ann Surg.

